# Jottings

**Published:** 1910-01-01

**Authors:** 


### JOTTINGS.

POUND day of the Hospital for Women, Brighton, was held last week. The institution is in need of funds.

THE directors of the London County and Westminster Bank, Limited, have voted a sum of 250 guineas to the special fund for the liquidation of the capital debt of St. Bartholomew's Hospital.

ON Saturday, December 18, the Marchioness of Donegal presided over the pantomime and Christmas-tree given at the National Hospital, Queen Square, by the members of the "Hearth and Home" Children's Circle.

THE offices of the special appeal committee of the Royal Waterloo Hospital for Children and Women have been removed to the premises of the hospital, Waterloo Road, S.E., where the work will be carried on as usual.

A LOCAL Government Board inquiry has been held into the application of the Cheshire Council for permission to borrow £82,000 for the extension of the Upton Pauper Lunatic Asylum. The asylum was built in 1829, and has been extended considerably since. Altogether £275,000 has been spent on it. The new annexe is to provide accommodation for 500 patients, of which 100 would be private patients.

MR. J. K. CAIRD has presented £10,000 to the Dundee Infirmary to enable the directors to take over the Sidlaw Infirmary.

THE Waverley Club recently gave an entertainment at the Cancer Hospital, Fulham Road, when "Lady Huntworth's Experiment" was performed. The comedy was capitally acted throughout and greatly enjoyed.

MR. THACKERAY TURNER, secretary of the Society for the Protection of Ancient Buildings, on writing to the Royal Commission on Historical Monuments (England) to inquire whether the Whitgift Hospital, Croydon, had been placed on the list of monuments decided by the Commission, in accordance with the terms of reference, to be most worthy of preservation, has received the following reply from the secretary: "The Commission have not yet begun their investigations in the county of Surrey, and therefore no list of 'selected' monuments in that county is as yet in existence. But at the same time I am to add that the Commission have good reason to believe that the hospital will be so included when the time comes, and they trust that some means may be found to secure its complete preservation."

